# Comparative Transcriptome Analysis of *Gleditsia sinensis* Thorns at Different Stages of Development

**DOI:** 10.3390/plants12071456

**Published:** 2023-03-27

**Authors:** Feng Xiao, Yang Zhao, Xiurong Wang, Yanan Sun

**Affiliations:** 1 Institute for Forest Resources and Environment of Guizhou/Key Laboratory of Forest Cultivation in Plateau Mountain of Guizhou Province/College of Forestry, Guizhou University, Guiyang 550025, China; maplexiao1994@163.com (F.X.); wxr7211@126.com (X.W.);; 2 Key Laboratory of Plant Resource Conservation and Germplasm Innovation in Mountainous Region (Ministry of Education), Guizhou University, Guiyang 550025, China; 3 Forestry Bureau of Xingren, Xingren 562300, China

**Keywords:** *Gleditsia sinensis* Lam., transcriptome, thorn, WGCNA

## Abstract

*G. sinensis* thorn (called “zào jiǎo cì”, ZJC) has important medicinal and economic value, however, little is known about the molecular mechanisms behind the development of ZJC. In this study, we measured the content of soluble sugar and starch during the growth and development of the thorn, and performed transcriptome sequencing of the thorn segment, non-thorn segment, apex, and root tip at five distinct stages of thorn formation. The results showed that, with the growth of ZJC, the soluble sugar content of the roots, hypocotyls, thorn stems, thornless stems, leaves, and the starch content of the roots and leaves all firstly increased and then decreased after the basic structure of thorns was formed; the soluble sugar content and starch content of ZJC showed an overall downward trend (decreased by 59.26% and 84.56%, respectively). *Myb-like*, *YABBY2*, *Growth-regulating factor 3*, *TCP2*, *Zinc transporter 8*, and another 25 genes may be related to the maintenance and growth of thorns. Gene Ontology (GO) enrichment analysis of differentially expressed genes (DEGs) between stems with thorn and thorn-free stems found that a significant number of DEGs were annotated with terms related to the positive regulation of development, heterochronic (GO:0045962), the positive regulation of photomorphogenesis (GO:2000306), and other biological process (BP) terms. The developmental initiation regulation of ZJC may be regulated by *TCP* transcription factors (TFs). Eight genes were selected randomly to validate the RNA-seq results using real-time quantitative PCR (RT-qPCR) and they indicated that the transcriptome data were reliable. Our work provided a comprehensive review of the thorn development of *G. sinensis*.

## 1. Introduction

*Gleditsia sinensis* Lam. (Fam.: *Caesalpinioideae*; Gen.: *Gleditsia*) is a widely distributed native tree species in China. *G. sinensis* thorn, known as “zào jiǎo cì” in Chinese (ZJC), is the main medicinal part of *G. sinensis*, which is rich in flavonoids, triterpenoid saponins, and other medicinal components [1,2]. There is a high demand for ZJC in China’s domestic market, but the adoption of improved cultivars is limited and there is considerable variability in the yield per plant. Therefore, research on the regulation of ZJC during critical stages of development is necessary.

Some epidermal structures of plants, such as trichomes, prickles, and thorns, deter various insects and herbivores [3]. Thorns are modified branches and spines are modified leaves, both containing adjoining vascular tissue, and prickles are formed by multiple cellular divisions of the epidermis and do not contain internal vascular tissue [4]. Trichomes in the seed form a coat of cotton, referred to as fibre, that has four continuous but overlapping developmental stages: initiation, elongation, secondary cell wall deposition, and maturation [5,6]. The trichomes’ initiation and patterning are under the control of the hub MYB-bHLH-WD40 in *Arabidopsis thaliana* [7]. The WD40 repeat protein TRANSPARENT TESTA GLABRA1 (TTG1), the R2R3 repeat MYB protein GLABRA1 (GL1), or WEREWOLF (WER), and the basic helix–loop–helix (bHLH) protein GLABRA3 (GL3), or ENHANCER OF GLABRA3 (EGL3), constitute an active R2R3 MYB-bHLH-WD40 complex that initiates trichome and non-hair cell differentiation [8]. Raspberry and rose prickles are modified glandular trichomes continuing to grow and eventually hardening into their final prickle morphologies [9]. The *Lycium ruthenicum* branch-thorns occur in response to drought and protect the corresponding axillary buds against drought stress [10]. Transcription factors such as MYB, bHLH, and TCP are involved in the development of prickles [11,12,13]. *TCP* TFs—*THORN IDENTITY1* (*TI1*) and *TI2*, are expressed in axillary meristems and bind to the promoter of WUSCHEL, repressing the maintenance of cell proliferation [14]. The disruption of the Citrus CEN (*CsCEN*) function results in the termination of the stem cell activity and the conversion of dormant axillary meristems into thorns [15]. Polar auxin transport in a thornless pummelo bud-sport may be responsible for the vigorous growth and thornless phenotype [16]. *G. sinensis* is branch thorn, originating from the meristem in the leaf axils, and it consists of the central zone (CZ), the peripheral zone (PZ), and the rib zone (RZ) composition. The thorn development of *G. sinensis* seedlings went through the thornless stage (2 d after germination, DAG), the thorn primordia stage (3 DAG), the basic completion stage of scale leaf (7 DAG), the thorn differentiation stage (8 DAG), and the basic thorn structure formation stage (14 DAG). Then, thorns begin to lignify (30 DAG), turn brown (75 DAG), and eventually turn completely brown (165 DAG) [17]. The concentration of 1 mg/L gibberellin can promote the growth of the trunk thorn and the weight of the per plant thorn in *G. sinensis* [18]. In total, the molecular mechanisms underlying the trichome or prickle development have been well studied; however, the molecular basis of thorn morphogenesis has been rarely studied and little is known about the molecular mechanisms behind the development of ZJC. The differential gene expression between thorn and non-thorn stems of *G. sinensis* may be associated with thorn induction, initiation, and development. In this study, to understand thorn development at the molecular levels, four different parts of *G. sinensis* at five developmental stages (2 DAG (labeled term A), 3 DAG (labeled term B), 7 DAG (labeled term C), 8 DAG (labeled term D), and 14 DAG (labeled term E)) were subjected to transcriptome sequencing (RNA-seq). The four different parts were the thorn stem segments (labeled S), the non-thorn stem segments (labeled U), the top of the stem (labeled T), and the tip of the root (labeled R), respectively.

## 2. Results

### 2.1. Changes in the Content of Carbohydrates during the Development of ZJC

**Figure 1:**
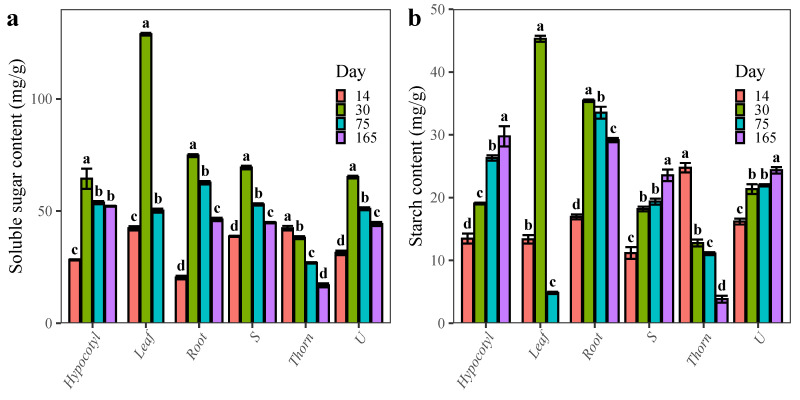
Changes in the physiological index content of ZJC development. (**a**): soluble sugar content; (**b**): starch content. Note: The data were analyzed by one-way analysis of variance (ANOVA) by R, different letters indicate the significant differences (*p* < 0.05, LSD) between means. The bars represent the mean ± SD. There was no leaf data because the leaves fall off when the seedlings of *G. sinensis* were 165d.

With the growth of *G. sinensis* seedlings, the soluble sugar content of roots, hypocotyls, thorny stems, thornless stems, and leaves initially increased and then decreased (Figure 1a). The starch content of hypocotyls, thorny stems, and thornless stems all showed an upward trend, while roots and leaves initially increased and then decreased, and ZJC showed an overall downward trend (Figure 1b). The soluble sugar content and starch content of *G. sinensis* thorn were the maximum when the basic structure of the thorn was formed (14 DAG), and reached the minimum when the thorn was completely browned (165 DAG). The soluble sugar content decreased by 59.26% and the starch content decreased by 84.56%.

### 2.2. Transcriptome Principal Component Analysis (PCA) and Quantitative Analysis of DEGs

**Figure 2:**
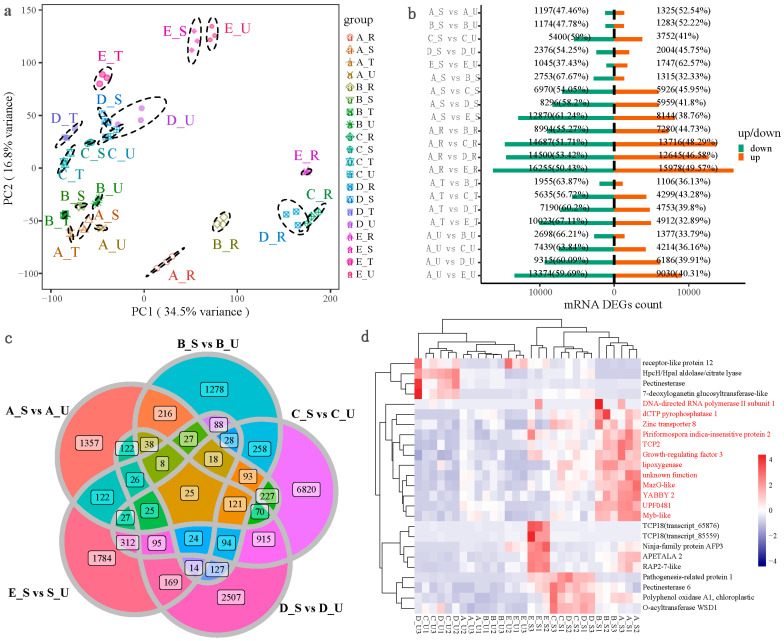
Sample PCA analysis and quantitative analysis of DEGs. (**a**): PCA analysis; (**b**): quantitative analysis of DEGs in different combinations; (**c**): venn analysis of different combinations; (**d**): heat map analysis of DEGs. Note: Different combinations in figure a were distinguished by color and shape, and the circles represented 95% confidence intervals; the texts in (**b**) were the number and proportion of up-regulated or down-regulated in one group.

A total of about 206 Gb clean reads were obtained after quality control. The Q30 of all samples were above 93.72%. PCA analysis results (Figure 2a) showed PC_1_ was 34.5%, PC_2_ was 16.8%, and the samples in the same group could be in tight aggregation. After screening for the DEGs, 1197 (47.46%) down-regulated genes and 1325 (52.54%) up-regulated genes existed in the A_S/A_U group; 1174 (47.78%) down-regulated DEGs and 1283 (52.22%) up-regulated DEGs existed in the B_S/B_U group; 5400 (59%) down-regulated DEGs and 3752 (41%) up-regulated DEGs existed in the C_S/C_U group; 2376 (54.25%) down-regulated DEGs and 2004 (45.75%) up-regulated DEGs existed in the D_S/D_U group; and 1045(37.43%) down-regulated DEGs and 1747 (62.57%) up-regulated DEGs existed in the E_S/E_U group (Figure 2b). Among the above five combinations, a total of 25 genes were collected between the thorn stem segments and the non-thorn stem segments (Figure 2c). Among them, in A_S and B_S, the relatively highly expressed genes were *Myb-like*, *YABBY2*, *Growth-regulating factor 3*, *TCP2*, *Zinc transporter 8*, etc. (Figure 2d).

### 2.3. Functional Enrichment Analysis of DEGs

**Figure 3:**
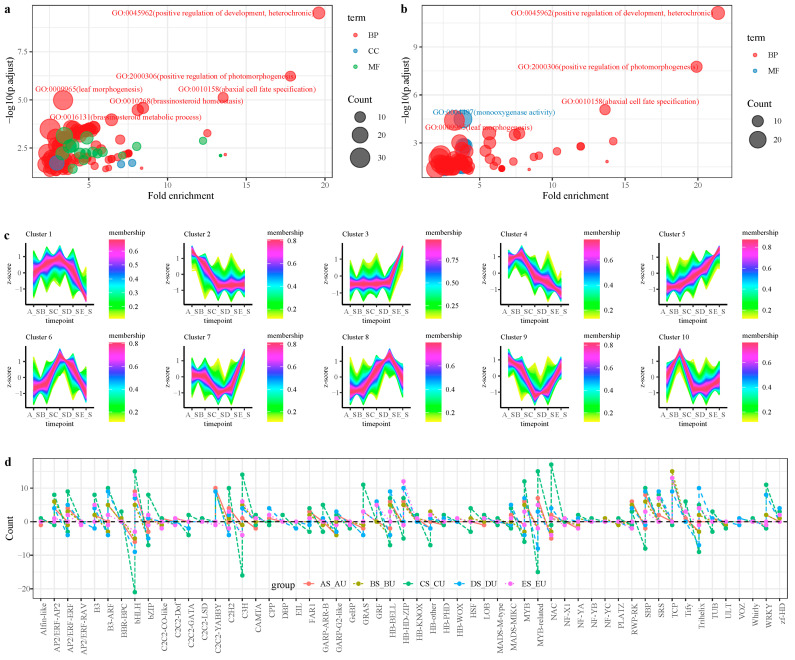
Sample PCA and correlation analysis. (**a**): GO enrichment analysis of DEGs in AS/AU group; (**b**): GO enrichment analysis of DEGs in BS/BU group; (**c**): Trend analysis of DEGs; (**d**): The differential expressed TFs in different group. Note: in (**a**), Fold enrichment = GeneRatio/BgRatio; in (**d**), the *X*-axis coordinates represent different TF families, the *Y*-axis represent the number of up-regulation or down-regulation, greater than 0 represents up-regulation number, and less than 0 represents down-regulation number.

Gene Ontology (GO) and the KEGG enrichment analysis of DEGs between different combinations demonstrated that, in the A_S/A_U group, the positive regulation of development, heterochronic (GO:0045962), the positive regulation of photomorphogenesis (GO:2000306), abaxial cell fate specification (GO:0010158), leaf morphogenesis (GO:0009965), floral organ morphogenesis (GO:0048444), adaxial/abaxial axis specification (GO:0009943), polarity specification of adaxial/abaxial axis (GO:0009944), and other biological process (BP) terms were significantly enriched (Figure 3a). KEGG enrichment analysis showed that Flavonoid biosynthesis (map00941), Phenylpropanoid biosynthesis (map00940), Linoleic acid metabolism (map00591), and other pathways were enriched in the A_S/A_U group. In the B_S/B_U group, positive regulation of development, heterochronic (GO:0045962), positive regulation of photomorphogenesis (GO:2000306), abaxial cell fate specification (GO:0010158), and other BP terms were significantly enriched (Figure 3b). Alpha-Linolenic acid metabolism (map00592), Brassinosteroid biosynthesis (map00905), Linoleic acid metabolism (map00591), Phenylpropanoid biosynthesis (map00940) were significantly enriched in the B_S/B_U group. In the C_S/C_U group, the regulation of auxin mediated signaling pathway (GO:0010928), auxin efflux (GO:0010315), auxin polar transport (GO:0009926), auxin transport (GO:0060918), the cellulose biosynthetic process (GO:0030244), and other BP terms were significantly enriched. In the D_S/D_U group, adaxial/abaxial axis specification (GO:0009943), the polarity specification of adaxial/abaxial axis (GO:0009944), auxin polar transport (GO:0009926), and other BP terms were significantly enriched. Alpha-Linolenic acid metabolism (map00592), Linoleic acid metabolism (map00591), ABC transporters (map02010), and Monoterpenoid biosynthesis (map00902) pathways were enriched in the D_S/D_U group. In the E_S/E_U group, triterpenoid metabolic process (GO:0006722), triterpenoid biosynthetic process (GO:0016104), monoterpene metabolic process (GO:0043692), tetracyclic triterpenoid metabolic process (GO:0010685), sesquiterpene metabolic process (GO:0051761), and other BP terms were significantly enriched. Flavonoid biosynthesis (map00941), Monoterpenoid biosynthesis (map00902), Cutin, suberine, and wax biosynthesis (map00073), Sesquiterpenoid and triterpenoid biosynthesis (map00909), and others pathways were enriched.

A trend analysis of DEGs in five different term combinations between the thorn and the non-thorn stem segments showed that the genes set of Cluster10 were significantly up-regulated in the B-S term, which was related to thorn primordia (Figure 3c), the GO enrichment analysis of Cluster10 was related to the positive regulation of the glycoprotein biosynthetic process, the positive regulation of protein glycosylation, and the induction of programmed cell death. The distribution of TFs in the DEGs of different combinations were analyzed (Figure 3d), the majority of *TCP* TFs were up-regulated in the thorn stem segments, the number of up-regulated gene in (AS vs. AU, BS vs. BU, CS vs. CU, DS vs. DU, ES vs. EU) was 13, 15, 13, 9, 13, respectively, and only transcript_1092 was down-regulated in CS vs. CU. In the AS/AU group, ten *YABBY* genes in AS were up-regulated. In the BS/BU group, *WUSCHEL-related homeobox 1* (transcript_138709, *WOX1*) was up-regulated in BS (log_2_FC = 2.3).

### 2.4. Weighted Co-Expression Network Analysis (WGCNA)

**Figure 4:**
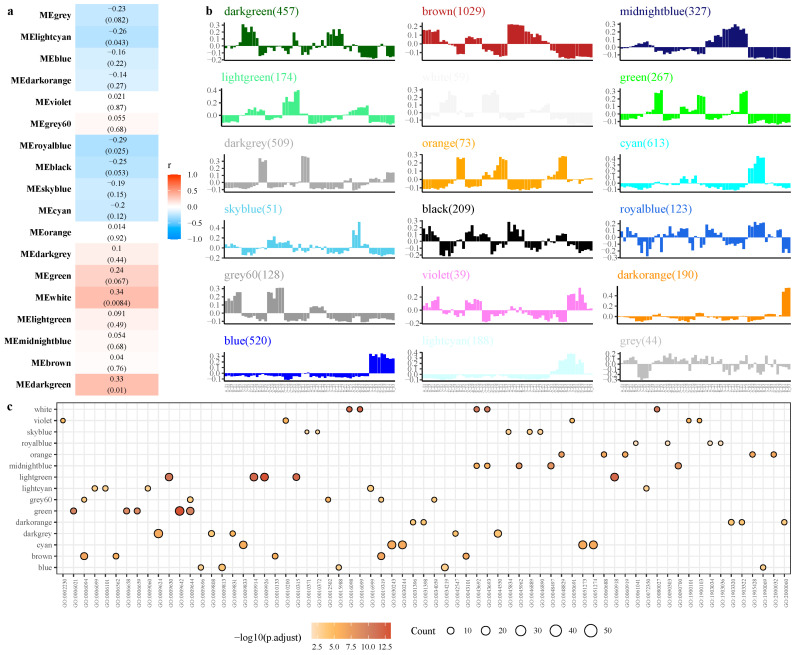
Weighted gene co-expression network analysis (WGCNA). (**a**): Module-trait correlation analysis; (**b**): The histograms described the eigengene expression of each module; (**c**): The GO enrichment analysis of each module (BP terms); Note: In (**a**), the trait values of the presence or absence of thorns were set to 1 for B-S, C-S, D-S, E-S, and 0 for other periods or parts. The color of each cell indicated the correlation coefficient between the module and traits (the top number in the cell represented the correlation coefficient, bottom one in parentheses represented the *p*-value). In (**b**), the *x*-axis indicates the samples, and the *y*-axis indicates the log_2_ “relative FPKM values” of the module eigengene. In (**c**), the top 5 BP terms of each combination filtered according to the *p*-value.

The analysis identified eighteen modules (labeled with diverse colors) (Figure 4a), of which the white module (containing 59 genes) had a weak positive correlation (*r*) of 0.34 (*p* = 0.0084), while the royal blue module had a weak negative correlation with trait (*r* = −0.29, *p* = 0.025). Monoterpenoid metabolic process (GO:0016098), monoterpenoid biosynthetic process (GO:0016099), and other BP terms were enriched in the white module. The top five significant GO-terms ranked by *p*-value for the BP in the midnight blue module were the maintenance of floral organ identity (GO:0048497), the maintenance of plant organ identity (GO:0090700), the positive regulation of development, heterochronic (GO:0045962), monoterpene metabolic process (GO:0043692), and monoterpene biosynthetic process (GO:0043693), and the genes in the midnight blue module were highly expressed mainly at the top of the stem (Figure 4b,c), while the genes in the blue, dark orange, and light cyan module were highly expressed mainly in the roots.

### 2.5. Verification of RNA-Seq Data

**Figure 5:**
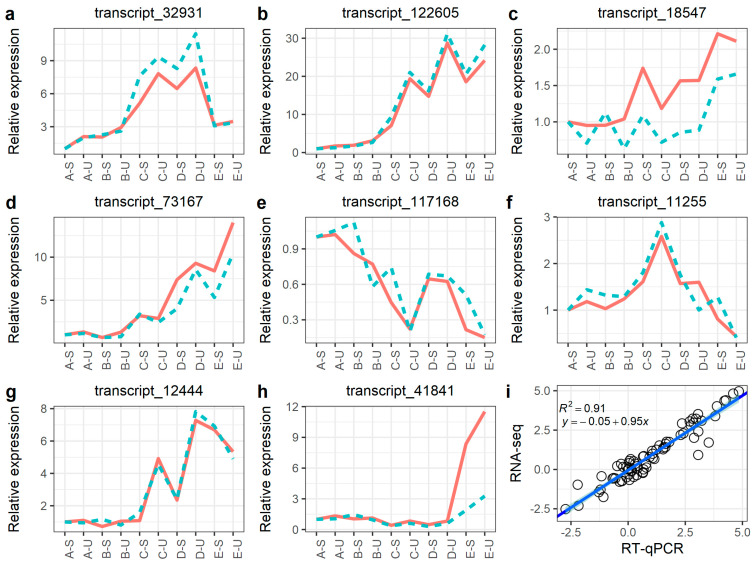
Correlation between the RT-qPCR and RNA-Seq data. (**a**–**h**): Relative expression of screened genes in transcriptome and RT-qPCR; (**i**): Correlation between the relative expression of transcriptome and RT-qPCR. Note: in (**a**–**h**), the blue line represented RT-qPCR, the red line represented RNA-seq.

To validate the accuracy of RNA-seq data, RT-qPCR was performed for eight genes randomly selected from DEGs. A strong positive correlation (*R*^2^ = 0.91) was obtained by a linear regression analysis (Figure 5i), suggesting that the transcriptome data were reliable.

## 3. Discussion

**Figure 6:**
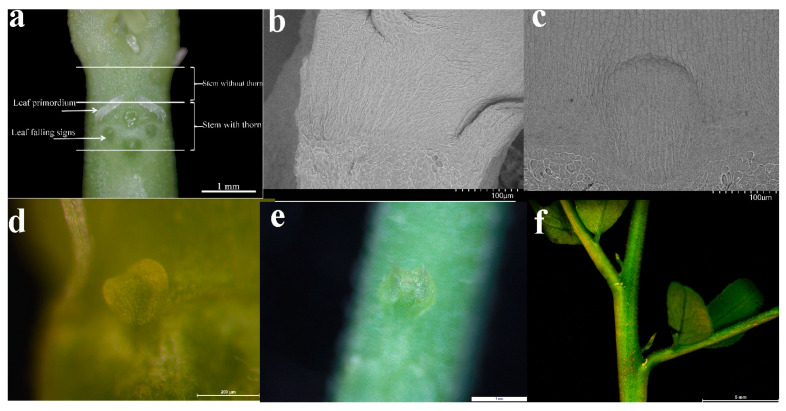
Demonstration diagram of test material collection. (**a**): Sampling stem section collection normal; (**b**): the thorn-free stage (2 DAG); (**c**): thorn primordium stage (3 DAG); (**d**): the stage of basic completion of scale leaves (7 DAG); (**e**): the thorn differentiation stage (8 DAG); (**f**): the thorn basic structure formation period (14 DAG).

Thorn primordia of *G. sinensis* appeared on the surface of the stem after 3 days of seed germination. Based on the morphological features of ZJC, five distinct development stages (A: thorn-free stage, B: thorn primordia stage, C: basically completed stage of scale leaves, D: thorn differentiation stage, and E: thorn basic structure formation stage) were identified and investigated (Figure 6b–f). With the growth of ZJC, the soluble sugar content of the roots, hypocotyls, thorn stems, thornless stems, leaves, and the starch content of the roots and leaves all firstly increased and then decreased after the basic structure of the thorns was formed. The starch content of hypocotyl, thorny stems, and thornless stems all increased gradually (Figure 1).

The RNA-seq can reflect the expression levels of genes in different physiological states of plants, revealing the molecular components of tissues and cells, and understanding development [19]. In order to reveal the molecular mechanism of ZJC, transcriptome sequencing at different stages of thorn development were performed, including the shoot apical meristem, root apical meristem, stem end with thorns, and stem without thorns. PCA analysis showed that the three replicate samples in each same group could be clustered together well, indicating that the accuracy of this sequencing result was high. Several notable developments and secondary metabolism-related TFs including *NAC*, *TCP*, *MYB*, *homeobox*, and *WRKY* were up-regulated in prickly internodes of *Rosa multiflora* [13]. A large number of TFs could be involved in the formation and development of trichomes, and the accumulation of *Rosa sterilis RsETC1* (*R3-MYB*) was significantly higher in inflorescence stems compared with pedicels [20]. A gradually increasing deposition of phenolic compounds and lignification with prickles development in *Rosa hybrida*, and secondary metabolism related genes such as the phenylpropanoid biosynthetic pathway were upregulated [21]. More than 60 compounds have been isolated and elucidated from the genus *Gleditsia*, including triterpenes, sterols, flavonoids, phenols, and alkaloids. Triterpenoid saponins are the most typical components of *Gleditsia* (not only found in pods, but also in thorns) [22,23]. A total of 457 secondary metabolites were detected in the *G. sinensis* and *G. microphylla* thorns, which were divided into eight categories: phenolic acids, alkaloids, terpenes, flavonoids, lignans, coumarins, tannins, and others [24]. In the D_S/D_U group, alpha-Linolenic acid metabolism (map00592), Linoleic acid metabolism (map00591), ABC transporters (map02010), and Monoterpenoid biosynthesis (map00902) pathways were enriched. In the E_S/E_U group, Flavonoid biosynthesis (map00941), Monoterpenoid biosynthesis (map00902), Cutin, suberine, and wax biosynthesis (map00073), Sesquiterpenoid and triterpenoid biosynthesis (map00909), and other pathways were enriched. The shoot tip’s strong demand for sugars inhibits axillary bud outgrowth by limiting the amount of sugar translocated to those buds [25]. The KEGG pathway of starch and sucrose metabolism is related to the occurrence of *Lycium ruthenicum* branch thorn [26]. With the growth and differentiation of ZJC, the synthesis genes related to secondary metabolism, such as flavonoids and terpenoids, were up-regulated, indicating that the flavonoids and terpenoids in the thorn-stem continued to accumulate.

GO enrichment analysis of DEGs between thorn and thorn-free stem found that a significant number of DEGs were related to the positive regulation of development, heterochronic (GO:0045962), the positive regulation of photomorphogenesis (GO:2000306), and other BP terms were significantly enriched (Figure 3a). *TCP2* (transcript_13023, transcript_117871, transcript_136554), *TCP4* (transcript_127617), *TCP13* (transcript_140199), *TCP4* (transcript_15920), and other *TCP* genes were enriched in the terms of GO:0045962. Shared DEGs in five periods of thorny and non-thorny stem segments showed that a total of 25 genes were identified, *Myb-like*, *YABBY2*, *Growth-regulating factor 3*, *TCP2*, and *Zinc transporter 8*, etc., were relatively highly expressed genes in A_S and B_S (Figure 2d). The majority of *TCP* TFs were up-regulated in the thorn-stem segments (Figure 3d). In previous studies, *TCP* TFs played an important role in prickle or trichome development. *Gossypium barbadense GbTCP* is an important transcription factor for fibre and root hair development [27]. *GsTCP12* and *CsTCP14* in *Citrus sinensis* may function in shoot branching, leaf development, or thorn development [28]. *TCP* is expressed in axillary meristems and binds to the promoter of *WUSCHEL*, repressing the maintenance of cell proliferation [29]. In the BS/BU group, *WUSCHEL-related homeobox 1* (transcript_138709, *WOX1*) was up-regulated in BS (log_2_FC = 2.3). This suggested that the developmental initiation regulation of ZJC may be regulated by *TCP* TFs.

We used the presence or absence of thorns in the sample (marked as 1 with stems, marked as 0 without thorns) as numerical values for co-expression network analysis. A few modules showed a weak positive/negative correlation; the white and royal blue modules had a weak positive correlation with the trait. The Monoterpenoid metabolic process (GO:0016098), the monoterpenoid biosynthetic process (GO:0016099), and other BP terms were enriched in the white module, indicating that the accumulation of terpenoids in ZJC begins when the thorn grows and differentiates.

## 4. Materials and Methods

### 4.1. Acquisition of Test Materials

The progeny seeds of *G. sinensis* semi-sibs were used as the test material, the seeds were subjected to germination treatment. The mixed soil in the pot was yellow soil: vermiculite: perlite soil (6:1:1) and was used as the cultivation substrate. The seedling management of watering and weeding was carried out in a unified manner, and the materials at the key time nodes in the development of ZJC were selected for sequencing, which were the thorn-free stage (2 DAG, labeled term A) (Figure 6b), the thorn primordia stage (3 DAG, labeled term B) (Figure 6c), the basically completed stage of scale leaves (7 DAG, labeled term C) (Figure 6d), the thorn differentiation stage (8 DAG after germination treatment, labeled term D) (Figure 6e), the thorn basic structure formation stage (14 DAG, labeled term E) (Figure 6f). The thorn stem segments (the stem segments about 1 mm above and below the leaf axils, labeled S) and the non-thorn stem segments (the remaining stem segments, labeled U) (Figure 6a) include the top of the stem (labeled T) and the tip of the root (labeled R). Fresh samples were observed by stereoscopic microscope and separated, immediately flash-frozen in liquid nitrogen, and stored at −80 °C until use; biology was repeated three times. In addition, *G. sinensis* roots, hypocotyls, thorny stem segments, thornless stem segments, leaves, and thorns at 14, 30, 75, 165 DAG were taken, the soluble sugar content and the starch content were measured according to the methods described in The Principle and Technology of Plant Physiology and Biochemistry Experiment Book [30].

### 4.2. RNA Extraction and Library Preparation

The RNA of *G. sinensis* samples were extracted using by Trizol reagent (Shenggong, Beijing, China) following the protocol. The RNA integrity was assessed by agarose gel electrophoresis while its integrity number (RIN) value was measured by Agilent 2100 (Agilent Technologies, Santa Clara, California, USA). The RNA extraction quality and concentration of all samples passed the inspection (A260/280 = 2.0~2.2; A260/230 = 1.8~2.2; 28S/18S = 1.4~2.7; Rin ≥ 8.0), the mRNA was enriched with Oligo (dT) magnetic beads. Fragmentation buffer was added to randomly fragment the mRNA into small fragments. Under the action of reverse transcriptase, one-strand cDNA was reversely synthesized using mRNA as a template, and then two-strand cDNA was synthesized. The double-strand cDNA was purified, end-repaired, A-tailed, and connected with sequencing adapters to establish the quality of the cDNA library. After passing the test, paired-end sequencing was performed on the Illumina Hiseq sequencing platform. The raw reads generated from Illumina sequencing have been deposited in the NCBI SRA database (accession BioProject: PRJNA869136).

### 4.3. Data Processing and Analysis

Using the Pacbio single molecule real time (SMRT) transcriptome of *G. sinensis* (accession BioProject: PRJNA722800) as the reference transcriptome, Diamond [31] was used to compare the background transcript to the NCBI NR, EuKaryotic Orthologous Groups (KOG), Gene Ontology (GO), Swiss-Prot, eggNOG, and Kyoto Encyclopedia of Genes and Genomes (KEGG) databases. The PlantTFDB database [32] was used to identify TFs. Using fastp [33] for quality control of raw dataaired-end reads from 60 libraries were mapped on the *G. sinensis* reference transcriptome by Bowtie2 [34]. RSEM [35] was used to obtain the read count and gene expression (FPKM, Fragments Per Kilobase of transcript per Million Mapped reads) value of each sample. PCA was performed using the expression levels of all samples. DESeq2 R package [36] was used to standardize the number of read counts in each sample (negative binomial distribution test (NB) was used to test the difference reads significance). Differentially expressed gene (DEG) screening threshold was set to *p*-value < 0.05 and |foldchange| > 2. The clusterProfiler R package was used to perform GO and KEGG enrichment of the DEGs [37]. DEGs clusters and visualize genes with similar expression patterns were performed by the Mfuzz R package [38], divided the DEGs into 10 clusters, the minimum score (membership) threshold was set to 0.25. Screening for the top 5000 genes of the gene expression matrix (background gene set for a union of DEGs between thorny and non-thorny stem segments in five terms) was conducted according to the median absolute deviation. The weighted co-expression network analysis (WGCNA) package [39] was performed to obtain the gene co-expression networks. Parameters were set up as power = 8, minModuleSize = 30, MEDissThres = 0.25.

### 4.4. Validation of the DEGs by Real-Time Quantitative PCR

Eight genes were selected randomly to validate the RNA-seq results using real-time quantitative PCR (RT-qPCR). The first strand of the cDNA fragment was synthesized from total RNA. RT-qPCR was performed on a real-time CFX96 Touch Real-Time PCR System (Bio-Rad, USA) with SYBR Green Real-time PCR Master reagents (Applied Biosystems, CA, USA). The PCR reaction conditions were as follows: preheating at 95 °C for 30 s, 40 cycles of heat denaturation at 95 °C for 5 s, annealing at 60 °C for 34 s. The gene relative expression levels were calculated from the threshold cycle according to the 2^−ΔΔCt^ method [40]. The detail of primers are shown in Supplemental Table S1, three technical replicates and three biology replicates were carried out, and the *G. sinensis actin* gene was used as the reference gene.

## 5. Conclusions

This study determined the levels of starch and soluble sugars in different stages and parts of ZJC development and analyzed the transcriptome during development. The results showed that starch and soluble sugar content in thorns were highest when the basic structure of the thorn was formed. Twenty-five genes in the comparisons between the thorn and non-thorn of *G. sinensis* at different stages may be related to the maintenance and growth of thorns. The initiation and regulation of ZJC development may be controlled by *TCP* TFs. RT-qPCR results validated the reliability of RNA-seq data. This study provides a solid foundation for understanding the initiation and developmental regulation of ZJC.

## Data Availability

The raw reads generated from Illumina sequencing have been deposited in the NCBI SRA database (accession BioProject: PRJNA869136).

## Supplementary Materials

The following supporting information can be downloaded at: https://www.mdpi.com/article/10.3390/plants12071456/s1, Table S1: Primer information of RT-qPCR.
