# Supplementary material for: Comparative Transcriptome Analysis of Gleditsia sinensis Thorns at Different Stages of Development
Source: Plants (Basel). 2023 Mar 27;12(7):1456. doi: 10.3390/plants12071456 (PMC10096692; doi:10.3390/plants12071456)
Supplement: Supplementary file 1 [file plants-12-01456-s001.zip › plants-2299762-supplementary.pdf]

Supplementary Materials

Table S1: Primer information of RT-qPCR

Primer Name	Sequences(5′→3′)
Reference Actin-F	GTGGTGGCTCAACTATGT
Reference Actin-R	TCCTCCAATCCAGACACT
transcript_11255-F	GCTCCTCCTCCTCCTAACC
transcript_11255-R	CCTTCCAATGCTCTGCTTCA
transcript_12444-F	TGTTGTGGAGGTGGAGAGT
transcript_12444-R	ATCGGAGCAGAAGCAAGTC
transcript_18547-F	AGAGGTGGTAGTTGAGGATGATTA
transcript_18547-R	ATCTGCTTGATACTTGGCTATGG
transcript_32931-F	AGGAGGAGTCAGTAAGCAAGAG
transcript_32931-R	TCCAACCAGCATCCAGTCA
transcript_41841-F	GTCAGAGGAGAGCAACTTGTG
transcript_41841-R	ACGGCGACAGTAGAACCA
transcript_73167-F	GAGAGCAACCTGACAACTATGAG
transcript_73167-R	GATGAACTGGACCACTGGAATC
transcript_122605-F	ATGAATGAGAGCAAGTTGATGGAT
transcript_122605-R	AGCAGCAGCAGCAGAAAG
transcript_117168-F	ATCACGGCTTAATCGCTCTG
transcript_117168-R	CAGTCATCGGCATTCCACAA
